# Cost-effectiveness study of therapeutic approaches for mucosal leishmaniasis

**DOI:** 10.1590/0102-311XEN132523

**Published:** 2024-08-19

**Authors:** Janaína de Pina Carvalho, Gláucia Cota, Mariana Lourenço Freire, Endi Lanza Galvão, Sarah Nascimento Silva, Tália Santana Machado de Assis

**Affiliations:** 1 Instituto René Rachou, Fundação Oswaldo Cruz, Belo Horizonte, Brasil.; 2 Universidade Federal dos Vales do Jequitinhonha e Mucuri, Diamantina, Brasil.; 3 Centro Federal de Educação Tecnológica de Minas Gerais, Contagem, Brasil.

**Keywords:** Mucocutaneous Leishmaniasis, Drug Therapy, Cost-Effectiveness Analysis, Leishmaniose Mucocutânea, Tratamento Farmacológico, Análise de Custo Efetividade, Leishmaniasis Mucocutánea, Tratamiento Farmacológico, Análisis de Costo-Efectividad

## Abstract

This study aimed to estimate the cost-effectiveness of four therapeutic approaches available for mucosal leishmaniasis in Brazil: miltefosine, meglumine antimoniate, combined with and without pentoxifylline, and liposomal amphotericin B. The perspective adopted was that of the Brazilian Unified National Health System (SUS). The outcome of interest was “cured patient”, which was analyzed using a decision tree model. Estimates of direct costs and effectiveness were obtained from the scientific literature. Meglumine antimoniate alone was the base comparator strategy; liposomal amphotericin B showed an incremental cost-effectiveness ratio (ICER) of USD 7,409.13 per cured patient, and the combination of meglumine antimoniate with pentoxifylline presented an ICER of USD 85.13. Miltefosine was absolutely dominated, with higher cost and similar effectiveness when compared to meglumine antimoniate. Sensitivity analyses, varying the cost by ±25%, did not change the results. However, when the cost of miltefosine was estimated at less than USD 171.23, this strategy was dominant over meglumine antimoniate alone. The results confirm that treatment with liposomal amphotericin B remains the option with the highest ICER among the approaches analyzed. Miltefosine may be cost-effective based on the variation in the acquisition price, which deserves attention because it is the only available oral option. The non-accounting of other aspects prevent the use of these results immediately to support decision-making, but they point out the need to negotiate the prices of drugs available for mucosal leishmaniasis and indicates the need of encouraging technology transfer or other actions aimed at expanding the performance of the Brazilian national industrial complex.

## Resumo

O objetivo deste estudo foi estimar o custo-efetividade de quatro abordagens terapêuticas disponíveis para leishmaniose mucosa no Brasil: miltefosina, antimoniato de meglumina, com e sem associação à pentoxifilina e anfotericina B lipossomal. A perspectiva adotada foi a do Sistema Único de Saúde (SUS). O desfecho de interesse foi “paciente curado”, que foi analisado por meio de um modelo de árvore de decisão. Estimativas de custos diretos e efetividade foram obtidas da literatura científica. O antimoniato de meglumina isolado foi a estratégia de comparação de base; a anfotericina B lipossomal apresentou razão de custo-efetividade incremental (RCEI) de USD 7.409,13 por paciente curado, e a combinação de antimoniato de meglumina com pentoxifilina apresentou RCEI de USD 85,13. A miltefosina foi dominada, com maior custo e efetividade semelhante quando comparada àquelas do antimoniato de meglumina. As análises de sensibilidade, variando o custo em ±25%, não alteraram os resultados. No entanto, quando o custo da miltefosina foi estimado em menos de USD 171,23, essa estratégia foi dominante sobre o antimoniato de meglumina isolado. Os resultados confirmam que o tratamento com anfotericina B lipossomal continua sendo a opção com maior RCEI entre as abordagens analisadas. Por sua vez, a miltefosina pode ser custo-efetiva com base na variação do preço de aquisição, o que merece atenção por ser a única opção oral disponível. A não consideração de outros aspectos impede o uso imediato desses resultados para subsidiar a tomada de decisão, mas apontam a necessidade de negociação dos preços dos medicamentos disponíveis para a leishmaniose mucosa e indicam a necessidade de incentivar a transferência de tecnologia ou outras ações que visem ampliar a atuação do complexo industrial nacional.

## Resumen

El objetivo de este estudio fue estimar el coste-efectividad de cuatro terapéuticas disponibles para la leishmaniasis mucosa en Brasil: miltefosina, antimoniato de meglumina, asociado con y sin pentoxifilina, y anfotericina B liposomal. La perspectiva adoptada fue la del Sistema Único de Salud brasileño (SUS). El resultado de interés fue el “paciente curado”, que se analizó mediante un modelo de árbol de decisión. Las estimaciones de costes directos y efectividad se obtuvieron de la literatura científica. El antimoniato de meglumina aislado fue la estrategia de comparación de referencia; el anfotericina B liposomal tuvo una razón coste-efectividad incremental (RCEI) de USD 7.409,13 por paciente curado, y la combinación de antimoniato de meglumina con pentoxifilina tuvo una RCEI de USD 85,13. Predominó la miltefosina, con un coste más elevado y una eficacia similar en comparación con la de antimoniato de meglumina. Los análisis de sensibilidad que variaron el coste en ±25% no alteraron los resultados. Sin embargo, cuando el coste de la miltefosina se estimó en menos de USD 171,23, esta estrategia resultó dominante sobre la antimoniato de meglumina aislada. Los resultados confirman que el tratamiento con anfotericina B liposomal sigue siendo la opción con el RCEI más elevado entre las terapéuticas evaluadas. A su vez, la miltefosina puede ser coste-efectiva en función de la variación del precio de compra, lo que merece atención al ser la única opción oral disponible. La falta de consideración de otros aspectos impide el uso inmediato de estos resultados para subvencionar la toma de decisiones, pero también apunta a la necesidad de negociar los precios de los fármacos disponibles para leishmaniasis mucosa y de fomentar la transferencia de tecnología u otras acciones dirigidas a ampliar el papel del sector industrial nacional.

## Introduction

Mucosal leishmaniasis predominates in neglected populations in the Americas, where approximately 1,500 cases occur per year ^1^. Despite the relatively small number of cases, compared to the cutaneous form, mucosal leishmaniasis can be considered the most severe form of cutaneous leishmaniasis due to its destructive and stigmatizing nature and potential to generate functional damage in the respiratory and digestive tracts ^2^.

Early diagnosis and appropriate treatment remain key strategies in the control of mucosal leishmaniasis. The treatment of 95% of patients diagnosed with the disease by 2030 was defined as a goal by the World Health Organization (WHO) ^3^. In this context, the availability of safe, cost-effective treatments that favor adherence and access are essential.

The current therapeutic recommendations of mucosal leishmaniasis are based on fragile scientific evidence and few options, mostly for parenteral use and with a high toxicity profile. In the Brazilian public health system, the recommended therapies are meglumine antimoniate, administered intramuscularly or intravenously (preferably combined with oral pentoxifylline); liposomal amphotericin B, intravenously ^4^; and oral miltefosine ^5^. The latter is the first oral medication available for the treatment of mucosal leishmaniasis and was incorporated into the Brazilian Unified National Health System (SUS) in 2018 ^5^ and made available in 2021 ^6^. All these therapeutic options require toxicity monitoring via periodic clinical and laboratory tests that may indicate the need for discontinuation (temporary or permanent) of treatment or even new intervention, to avoid further damage to health. The most worrying adverse events for meglumine antimoniate are cardiac disorders and the most common are musculoskeletal disorders (such as arthralgias and myalgias) and hepatic, pancreatic, or renal alterations. Infusion reactions are the most frequently reported events with liposomal amphoterecin and kidney disorders (especially an increase in creatinine), which are the events that require the greatest caution. The main concern for miltefosine is its teratogenic potential and the most frequently observed events are gastrointestinal disorders (such as nausea and vomiting) ^4^ ^,^ ^7^ ^,^ ^8^.

The SUS is one of the largest and most complex public health systems in the world, guaranteeing full, universal, and free access to the entire population of the country. To ensure the population’s access to appropriate technologies in a sustainable manner, it is necessary to adopt an evidence-based decision-making process ^9^ ^,^ ^10^. In Brazil, the process of incorporating new health technologies in the SUS includes an evaluation by the Brazilian National Commission for the Incorporation of Health Technologies (CONITEC) and is based on explicit criteria developed in the field of health technology evaluations to guide decision-making. Ultimately, the parameters to be considered are, among others, the effectiveness, safety, and costs of the technology ^11^.

As part of the dossier for miltefosine’s incorporation into the SUS, the cost-effectiveness analysis conducted by the CONITEC was based on studies involving patients with the cutaneous form of leishmaniasis ^5^. However, no complete economic evaluation for the specific treatment of mucosal leishmaniasis was identified in the official documents or in the scientific literature. Therefore, the objective of this study was to perform an economic analysis of the therapeutic approaches available for mucosal leishmaniasis in Brazil.

## Methods

### Study design

This economic study was designed as a cost-effectiveness analysis aiming to compare four different therapeutic approaches for mucosal leishmaniasis available in Brazil: miltefosine, meglumine antimoniate (combined with and without pentoxifylline), and liposomal amphoterecin. The perspective adopted was that of the payer, the SUS, and the time horizon comprised the beginning of treatment until consultation for outcome evaluation (six months after treatment).

The target population considered in this cost-effectiveness analysis was the annual average of confirmed cases of mucosal leishmaniasis in Brazil, considering that all cases of mucosal leishmaniasis were treated with each of the approaches and excluding cases with contraindications for use (as detailed by Carvalho et al. ^12^). The number of patients was obtained by Carvalho et al. ^12^ to estimate costs for treatment based on the mucosal leishmaniasis cases reported to the Brazilian Information System for Notificable Diseases (SINAN, acronym in Portuguese) from 2014 to 2018, totaling 1,075 cases. Currently (considering the period from 2018 to 2022), the average number of cases has decreased to 975 cases per year, most of them are men (76%) and over 20 years old (90% of cases) ^13^. This study followed the Brazilian Ministry of Health methodological guidelines for economic evaluations ^14^.

### Details of the therapeutic approaches evaluated

(a) Miltefosine (50mg per capsule): body weight < 45kg - 100mg/day for 28 days; body weight > 45kg - 150mg/day for 28 days;

(b) Meglumine antimoniate (5mL per ampoule (81mgSb+5/mL)): 20mg/kg/day (up to a maximum of 1,215mg or 3 ampoules) for 30 days;

(c) Meglumine antimoniate (as described above) combined with pentoxifylline - 400mg per film-coated tablet every 8 hours for 30 days; and

(d) Liposomal amphotericin B (50mg per ampoule): 3-5mg/kg/day (up to a cumulative total of 25-40mg/kg) for approximately 10 days.

### Cost and effectiveness

The direct cost of the therapeutic approaches has been estimated by Carvalho et al. ^12^ using the macrocosting technique based on the combination of expenses arising from mucosal leishmaniasis or adjuvant drugs and those indicated for contraception, in addition to costs for procedures performed by the health team and for complementary exams (Table 1). Costs related to diagnosis were not included since they were out of the scope of this analysis. On the other hand, although costs associated with adverse events could contribute to differentiate the mucosal leishmaniasis therapies, as shown by Carvalho et al. ^8^ in an extensive literature review, the lack of consistent data on the incidence of adverse events prevented the estimation of costs arising from toxicity. The treatment costs were primarily estimated using the values from January 2019 as reference ^12^, then updated based on the official inflation rate in December 2023, determined by cumulative the Extended National Consumer Price Index (IPCA, acronym in Portuguese), with a correction index of 1.33, corresponding to 32.8% (from January 2019 to December 2023) ^15^. All costs are reported in US dollars (USD) with a conversion rate for December 2023 of 1 USD = 4.8407 Brazilian Reais (BRL) ^16^.

**Table 1:** Cost components, direct costs, and effectiveness of the therapeutic approaches evaluated for mucosal leishmaniasis in Brazil.

Therapeutic approach	Cost components considered (% of direct costs)	Average total cost of treatment (USD) [variation of ± 25%, base year 2023]	Effectiveness/Cure rate
	% (95%CI)	% (95%CI)
Miltefosine	Drug (87.5) Contraception (0.9) Procedure: 6 medical visit in specialized care (5.9) Complementary tests: renal and liver function and beta HCG (5.8)	281.29 (210.97-351.61)	65.2 (56.4-73.0)
Meglumine antimoniate	Drug (67.0) Procedure: 6 medical visit in specialized care + 30 administration of drugs in specialized care (13.0) Complementary tests: cardiac monitoring, hematopoietic function, renal, liver, and pancreatic function, serum electrolytes, and beta HCG (21.0)	171.44 (128.58-214.30)	65.1 (52.8-75.6)
Meglumine antimoniate + pentoxifylline	Drug (63.0) Adjuvant (pentoxifylline) (6.0) Procedure: 6 medical visit in specialized care + 30 administration of drugs in specialized care (12.0) Complementary tests: cardiac monitoring, hematopoietic function, renal, liver and pancreatic function, and beta HCG (20.0)	181.45 (136.09-226.82)	77.4 (51.4-91.7)
Liposomal amphotericin B	Drug (90.0) Procedure: 2 medical visit in specialized care + 1 treatment of other diseases due to protozoa - hospitalization + 5 daily cost of stay above the hospitalization standard length (9.0) Complementary tests: hematopoietic function, renal and liver function, serum electrolytes, and beta HCG (1.0)	774.18 (580.64-967.73)	85.2 (75.8-91.3)

95%CI: 95% confidence interval.

Source: adaptation from Carvalho et al. ^8^ and Carvalho et al. ^12^.

Effectiveness, assumed as the cure rate, was estimated via a comprehensive systematic review conducted by Carvalho et al. ^8^. Considering the lack of randomized controlled trials (RCT) addressing the efficacy of different therapeutic interventions for mucosal leishmaniasis, data from observational non comparative studies were gathered covering the accumulated experience in the treatment of mucosal leishmaniasis in the Americas. In total, 27 studies were included, most of them conducted in Brazil (17 studies). The quality of the studies was assessed with design-specific tools and, in general, considered poor, with great variation in the criteria adopted for cure assessment. Despite heterogeneous, in most studies, cure was assumed as the complete epithelialization of all lesions, associated with the disappearance of inflammatory signs (infiltration, edema, redness) and was assessed within one year after the end of treatment. Despite the limitations, the results presented for cure rates for different therapies converge with those estimated in RCTs and present estimates for therapies for which RCTs have not yet been conducted.

### Models used in the cost-effectiveness and sensitivity analyses

**Figure 1:**
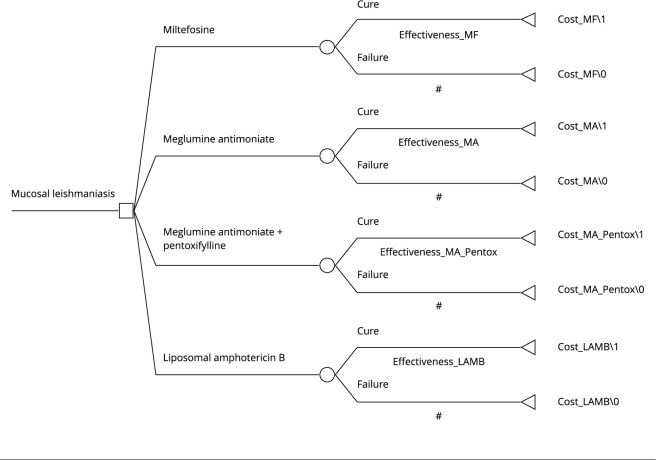
Basic structure of the decision tree used to compare the four treatment approaches.

LAMB: liposomal amphotericin B; MA: meglumine antimoniate; MF: miltefosine; Pentox: pentoxifylline.

The cost-effectiveness analysis was conducted using a decision tree model and TreeAge Pro Healthcare, 2022 R1.2 software (https://www.treeage.com/). In the analysis, the outcome of interest was “cured patient”. The incremental cost-effectiveness ratio (ICER) was defined as the proportion of the difference in cost of the alternatives to the difference in effectiveness. Initially, an analysis was performed comparing the four treatment approaches of interest (Figure 1).

Univariate deterministic sensitivity analyses were conducted using the tornado diagrams technique, combining the different therapeutic approaches in pairs and varying the parameters in one hundred ranges of intervals, to verify the influence of uncertainty on the main model parameters (cost and effectiveness). Direct costs arbitrarily varied by ±25%. Regarding effectiveness, the influence of variation in cure rates was explored considering the 95% confidence interval (95%CI) estimated in the systematic review for each treatment approaches, as presented in Table 1. An exploratory sensitivity analysis was also conducted, varying the miltefosine costs to zero and effectiveness up to 100%, to identify the value at which this approach would become cost-effective.

## Results

Based on the cost-effectiveness analysis of the four treatment approaches, the ICERs were USD 7,409.13 and USD 83.42 per case of mucosal leishmaniasis cured with liposomal amphoterecin and meglumine antimoniate + pentox, respectively. Miltefosine was found to be absolutely dominated, that is, presented a higher cost with similar effectiveness to those for meglumine antimoniate alone (Table 2).

**Table 2:** Cost-effectiveness analysis of four treatment approaches for mucosal leishmaniasis.

Therapeutic approach	Cost (USD)	Incremental cost (USD)	Effectiveness	Incremental effectiveness	ICER	Dominance
Meglumine antimoniate	171.44		0.65			Undominated
Meglumine antimoniate + pentoxifylline	181.45	10.01	0.77	0.12	83.42	Undominated
Miltefosine	281.29	99.84	0.65	-0.12	-832.00	Absolutely dominated
Liposomal amphotericin B	774.18	592.73	0.85	0.08	7,409.13	Undominated

ICER: incremental cost-effectiveness ratio.

**Figure 2:**
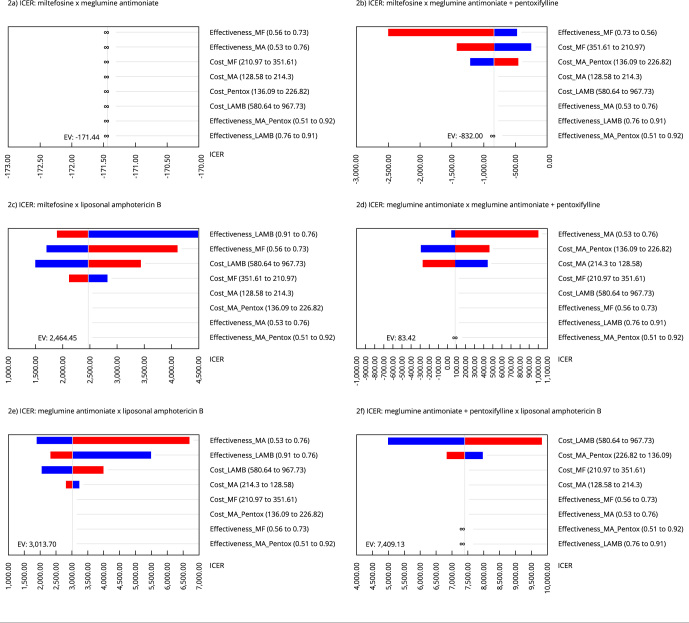
Tornado diagrams of deterministic sensitivity analyses combining the different therapeutic approaches in pairs.

ICER: incremental cost-effectiveness ratio; EV: expected value; LAMB: liposomal amphotericin B; MA: meglumine antimoniate; MF: miltefosine; Pentox: pentoxifylline.

The deterministic sensitivity analyzes carried out using tornado diagrams can be seen in Figure 2 and are represented in Table 3. These analyses allow verifying the individual impact of the cost and effectiveness variables on the ICER according to the different pairs of therapeutic approaches: (a) miltefosine x meglumine antimoniate; (b) miltefosine x meglumine antimoniate + pentox; (c) miltefosine x liposomal amphoterecin; (d) meglumine antimoniate x meglumine antimoniate + pentox; (e) meglumine antimoniate x liposomal amphoterecin; (f) liposomal amphoterecin x meglumine antimoniate + pentox.

**Table 3:** Results of the deterministic sensitivity analysis of the impact on the incremental cost-effectiveness ratio (ICER) combining the different therapeutic approaches in pairs.

Pair of therapeutic approach (dominance or ICER)/Variable description	Low	Base	High	Impact	Low ICER	High ICER	Spread
Miltefosine (dominated) x meglumine antimoniate (0)							
Effectiveness_MF	0.56	0.65	0.73	Increase	-21,970	292,933,333	∞
Effectiveness_MA	0.53	0.65	0.76	Increase	-209,238,095	21,970	∞
Cost_MF	210.97	281.29	351.61	Increase	0	0	∞
Cost_MA	128.58	171.44	214.3	Increase	0	0	∞
Cost_MA_Pentox	136.09	181.45	226.82	Increase	0	0	∞
Cost_LAMB	580.64	774.18	967.73	Increase	0	0	∞
Effectiveness_MA_Pentox	0.51	0.77	0.92	Increase	0	0	∞
Efffectiveness_LAMB	0.76	0.85	0.91	Increase	0	0	∞
Miltefosine (dominated) x meglumine antimoniate + pentoxifylline							
Effectiveness_MF	0.56	0.65	0.73	Decrease	-2,496	-47,542,857	202,057,143
Cost_MF	210.97	281.29	351.61	Decrease	-1,418	-246	1,172
Cost_MA_Pentox	136.09	181.45	226.82	Increase	-1,210	-45,391,667	75,608,333
Cost_MA	128.58	171.44	214.3	Increase	-832	-832	0
Cost_LAMB	580.64	774.18	967.73	Increase	-832	-832	0
Effectiveness_MA	0.53	0.65	0.76	Increase	-832	-832	0
Effectiveness_LAMB	0.76	0.85	0.91	Increase	-832	-832	0
Effectiveness_MA_Pentox	0.51	0.77	0.92	Increase	-1,536	2,662.4	∞
Miltefosine (undominated) x liposomal amphotericin (2,464.45)							
Effectiveness_LAMB	0.76	0.85	0.91	Decrease	189,573,077	448,081,818	258,508,741
Effectiveness_MF	0.56	0.65	0.73	Increase	169,962,069	410,741,667	240,779,598
Cost_LAMB	580.64	774.18	967.73	Increase	1,496.75	3,432.2	1,935.45
Cost_MF	210.97	281.29	351.61	Decrease	2,112.85	2,816.05	703.2
Cost_MA	128.58	171.44	214.3	Increase	2,464.45	2,464.45	0
Cost_MA_Pentox	136.09	181.45	226.82	Increase	2,464.45	2,464.45	0
Effectiveness_MA	0.53	0.65	0.76	Increase	2,464.45	2,464.45	0
Effectiveness_MA_Pentox	0.51	0.77	0.92	Increase	2,464.45	2,464.45	0
Meglumine antimoniate (undominated) x meglumine antimoniate + pentoxifylline (83.42)							
Effectiveness_MA	0.53	0.65	0.76	Increase	41,708,330	1,001	959,291,670
Cost_MA_Pentox	136.09	181.45	226.82	Increase	-294,5830330	461.5	756,083,330
Cost_MA	128.58	171.44	214.3	Decrease	-273.75	44,058,333	714,333.330
Cost_MF	210.97	281.29	351.61	Increase	83,416,670	83,416,670	0
Cost_LAMB	580.64	774.18	967.73	Increase	83,416,670	83,416,670	0
Effectiveness_MF	0.56	0.65	0.73	Increase	83,416,670	83,416,670	0
Effectiveness_LAMB	0.76	0.85	0.91	Increase	83,416,670	83,416,670	0
Effectiveness_MA_Pentox	0.51	0.77	0.92	Increase	-266,933,330	154	∞
Meglumine antimoniate (undominated) x liposomal amphotericin (3,013.70)							
Effectiveness_MA	0.53	0.65	0.76	Increase	18,835,625	669,711,111	481,354,861
Effectiveness_LAMB	0.76	0.85	0.91	Decrease	231,823,077	547,945,455	316,122,378
Cost_LAMB	580.64	774.18	967.73	Increase	2,046	3,981.45	1,935.45
Cost_MA	128.58	171.44	214.3	Decrease	2,799.4	3,228	428.6
Cost_MF	210.97	281.29	351.61	Increase	3,013.7	3,013.7	0
Cost_MA_Pentox	136.09	181.45	226.82	Increase	3,013.7	3,013.7	0
Effectiveness_MF	0.56	0.65	0.73	Increase	3,013.7	3,013.7	0
Effectiveness_MA_Pentox	0.51	0.77	0.92	Increase	3,013.7	3,013.7	0
Liposomal amphotericin (undominated) x meglumine antimoniate + pentoxifylline (7,049.13)							
Cost_LAMB	580.64	774.18	967.73	Increase	4,989,875	9,828.5	4,838,625
Cost_MA_Pentox	136.09	181.45	226.82	Decrease	6,842	7,976,125	1,134,125
Cost_MF	210.97	281.29	351.61	Increase	7,409,125	7,409,125	0
Cost_MA	128.58	171.44	214.3	Increase	7,409,125	7,409,125	0
Effectiveness_MF	0.56	0.65	0.73	Increase	7,409,125	7,409,125	0
Effectiveness_MA	0.53	0.65	0.76	Increase	7,409,125	7,409,125	0
Efectiveness_MA_Pentox	0.51	0.77	0.92	Increase	-846,757,143	1,823,784,615	∞
Effectiveness_LAMB	0.76	0.85	0.91	Increase	-59,273	2,155,381,818	∞

LAMB: liposomal amphotericin B; MA: meglumine antimoniate; MF: miltefosine; Pentox: pentoxifylline.

As miltefosine x meglumine antimoniate (a) exhibit similar cost and effectiveness profiles, no dominancy is expected in the cost-effectiveness analysis. In the case of miltefosine x meglumine antimoniate + pentox (b), the pivotal factors affecting ICER is the effectiveness followed by cost of miltefosine. In the comparison miltefosine x liposomal amphoterecin (c), the main factor affecting ICER is the effectiveness of both drugs, but costs also have a considerable impact. On the other hand, in the analysis involving the approaches meglumine antimoniate x meglumine antimoniate + pentox (d), cost is the main factor. Comparing meglumine antimoniate x liposomal amphoterecin (e), the difference in effectiveness between the treatments emerges as the most influential factor on the ICER. Lastly, for liposomal amphoterecin x meglumine antimoniate + pentox (f), the effectiveness of the approaches is irrelevant to the ICER and the cost of liposomal amphoterecin is the determining factor affecting the ICER.

The univariate deterministic sensitivity analyses using a hundred ranges of variations indicated that, when the cost of meglumine antimoniate alone was ≥ USD 181.73, this therapeutic option and miltefosine were absolutely dominated. When the cost of meglumine antimoniate combined with pentoxifylline was ≤ USD 170.57, the option of meglumine antimoniate alone became absolutely dominated. The exploratory analysis of miltefosine cost indicated that, when the cost of this approach was ≤ USD 171.23, this therapeutic option became the comparator strategy, with meglumine antimoniate alone becoming completely dominated, showing an ICER of USD 7,409.13 for liposomal amphoterecin and USD 85.13 for meglumine antimoniate + pentox.

By varying the effectiveness of miltefosine and meglumine antimoniate alone, miltefosine remained absolutely dominated. Miltefosine was only cost-effective if the cure rate was greater than 79% (variation greater than the 95%CI). With regard to meglumine antimoniate + pentox, a reduction in effectiveness to ≤ 65% would make it absolutely dominated, and an increase of ≥ 85% would make liposomal amphoterecin completely dominated. The latter would also be completely dominated if its effectiveness was reduced to ≤ 77%.

## Discussion

The availability of cost-effective therapeutic strategies for mucosal leishmaniasis is a challenge since it is a neglected tropical disease that has received little investment in research and public policies, culminating in the current scenario of few and suboptimal therapeutic options ^7^. In this sense, the exploration of new and old treatment options for mucosal leishmaniasis, including comprehensive parameters aligned with the principles of health technology evaluations, emerges as an interesting path in the search for more appropriate interventions for the management of leishmaniasis.

Recommendations related to the willingness-to-pay threshold in Brazil are recent. The Brazilian Ministry of Health recommends that technology assessments adopt a reference parameter and that quality-adjusted years of life (QALY) be used as the main outcome. In this case, a cost-effective technology is considered to be one that does not exceed the value of BRL 40,000.00 (or USD 8,263.27) per QALY and, in alternative situations, a variation of 3x this value is accepted ^17^. Mucosal leishmaniasis is included in these alternative situations, as they are endemic diseases in low-income populations and with few therapeutic alternatives available; however, the QALY value for this disease is not yet available in the literature. Even so, if we consider the outcome of this study (the cure rate), as an approximation of the QALY result, all the approaches could be considered cost-effective, as none of the ICERs exceeded the established limit of USD 8,263.27 (the highest ICER identified was that of liposomal amphoterecin, with a value of USD 7,409.13).

Moreover, although effectiveness stands out as the most likely variable to impact the ICER across different scenarios, it is generally considered a non-manipulable characteristic. In this sense, considering that cure rates were derived from non-randomized trials (nRCT), which adds a significant potential for bias, it is important to state that, except for a study evaluating miltefosine, in which high-quality design (RCT) with longer follow-up time showed greater efficacy than indirect comparisons using pooled rates, no significant changes are expected in the findings. Therefore, potential cost variations resulting from different strategies (external dependence, number of producers, negotiation capacity, among others) emerge as the most feasible alternatives to influence the cost-effectiveness of the therapeutic approaches presented.

Although miltefosine was dominated by the other therapeutic options in this study, it is a drug with some characteristics that may represent potential advantages in a decision-making algorithm, especially its oral use and few absolute restrictions on its use. The greater convenience in dosage, with the possibility of administration at home and follow-up on an outpatient basis, may represent factors that facilitate access, influencing patient adherence. However, more studies are still needed to attest to the efficacy and tolerability of this drug in different populations and on a large scale, given the scarce knowledge on mucosal leishmaniasis and the specificities related to the safety profile of the drug ^18^. Another important point to note is the observation, at this time, of similarity in the effectiveness observed for miltefosine and meglumine antimoniate alone but the significant difference in the cost parameter (difference of USD 110.00 per treatment with miltefosine).

Considering the aforementioned information, it is unknown to the extent to which the use of an oral medication, with the potential to reduce the impact on quality of life and absenteeism, overcomes the incremental cost generated by its use when compared to meglumine antimoniate. In this context, we highlight that our study did not include nonmedical direct costs, such as patient expenses for transportation and food, which are expected in the context of mucosal leishmaniasis treatment.

We also found that a reduction in the purchase value of miltefosine at an average treatment cost of less than USD 171.23 makes this approach the comparator strategy, with meglumine antimoniate alone absolutely dominated, and meglumine antimoniate + pentox presenting an ICER of USD 85.13. The highest incremental cost per case cured for liposomal amphoterecin (USD 7,409.13), which is more effective than the others, should be a focus on negotiations involving the acquisition of medications. This observation confirms the importance of actions subsequent to the incorporation of technologies into the SUS, in this case, negotiations for the acquisition of health technologies. Currently, the centralized acquisition of drugs for the leishmaniasis program in Brazil involves a strategy to increase purchasing power and promote a reduction in the final price. In addition, in the context of a neglected disease, negotiations involving international organizations allow for a differentiated understanding of this trade relationship based on the principle of social responsibility and joint effort to achieve global goals already agreed upon by the WHO for this decade ^3^.

Factors related to the robustness of the analyses presented here should be highlighted, including the use of updated and estimated parameters based on real scenarios of use of the technologies of interest, that is, within the scope of the SUS. This is the first economic analysis available in the literature that focuses on the treatment of mucosal leishmaniasis. As a limitation, only direct medical costs were accounted for; nonmedical direct costs, as well as indirect and intangibles costs were not considered in the analysis. Moreover, our analysis presented a simple analytical model that does not add results related to adverse events from the therapies used, which could impact the total costs of treatments. We highlight, however, that reliable data are still scarce to estimate the true frequency of adverse events ^8^ and, thus, more robust studies are needed to explore this result in subsequent analyses. The cure rates adopted herein as measures of effectiveness are also a limitation, as they are an approximation of reality due to being estimated via a systematic review of studies with many methodological weaknesses ^10^. Finally, economic analyses of mucosal leishmaniasis are intrinsically complex since they involve nonbinary decision scenarios, subject to multiple parameters influencing the decisions. Thus, this is a clinical situation in which there will hardly be a single therapeutic option indicated for all cases but eligible therapeutic options for a given subgroup of patients, which complicates the projection of costs. However, the sensitivity analysis carried out in this study, added to the results of Carvalho et al. ^12^ (as summarized in Table 1) is useful for identifying the items with the greatest participation or impact on the costs generated by each therapeutic intervention for mucosal leishmaniasis, allowing an analysis of the potential for intervention and, if applicable, the planning of specific actions to minimize costs.

In summary, the data indicate that the cost of drug acquisition is the main component of the total expenditure on mucosal leishmaniasis treatment, indicating a need to review the negotiation process involved in drug purchases. The fact that almost all available drugs are produced by a single manufacturer outside Brazil is noteworthy, reinforcing the importance of encouraging technology transfer and other actions aimed at expanding the performance of the national industrial complex. These data represent the first step toward therapeutic decisions for mucosal leishmaniasis based on cost-effectiveness criteria.
